# ‘I Just Stopped Going’: A Mixed Methods Investigation Into Types of Therapy Dropout in Adolescents With Depression

**DOI:** 10.3389/fpsyg.2019.00075

**Published:** 2019-02-05

**Authors:** Sally O’Keeffe, Peter Martin, Mary Target, Nick Midgley

**Affiliations:** ^1^Research Department of Clinical, Educational and Health Psychology, University College London, London, United Kingdom; ^2^Child Attachment and Psychological Therapies Research Unit, Anna Freud National Centre for Children and Families, London, United Kingdom; ^3^Department of Applied Health Research, University College London, London, United Kingdom

**Keywords:** attrition, dropout, premature termination, psychotherapy, adolescents, depression, mixed-methods, ideal type analysis

## Abstract

What does it mean to ‘drop out’ of therapy? Many definitions of ‘dropout’ have been proposed, but the most widely accepted is the client ending treatment without agreement of their therapist. However, this is in some ways an external criterion that does not take into account the client’s experience of therapy, or reasons for ending it prematurely. This study aimed to identify whether there were more meaningful categories of dropout than the existing dropout definition, and to test whether this refined categorization of dropout was associated with clinical outcomes. This mixed-methods study used a subset of data from the IMPACT trial, which investigated psychological therapies for adolescent depression. Adolescents were randomly allocated to a treatment arm (Brief Psychosocial Intervention; Cognitive-Behavioral Therapy; Short-Term Psychoanalytic Psychotherapy). The sample for this study comprised 99 adolescents, aged 11–17 years. Thirty-two were classified as having dropped out of treatment and participated in post-therapy qualitative interviews about their experiences of therapy. For 26 dropout cases, the therapist was also interviewed. Sixty-seven cases classified as having completed treatment were included to compare their outcomes to dropout cases. Interview data for dropout cases were analyzed using ideal type analysis. Three types of dropout were constructed: ‘dissatisfied’ dropout, ‘got-what-they-needed’ dropout, and ‘troubled’ dropout. ‘Dissatisfied’ dropouts reported stopping therapy because they did not find it helpful. ‘Got-what-they-needed’ dropouts reported stopping therapy because they felt they had benefitted from therapy. ‘Troubled’ dropouts reported stopping therapy because of a lack of stability in their lives. The findings indicate the importance of including the perspective of clients in definitions of drop out, as otherwise there is a risk that the heterogeneity of ‘dropout’ cases may mask more meaningful distinctions. Clinicians should be aware of the range of issues experienced by adolescents in treatment that lead to disengagement. Our typology of dropout may provide a framework for clinical decision-making in managing different types of disengagement from treatment.

## Introduction

Dropout from psychological treatment is a significant concern across mental health services, including services for children and young people. The study of dropout has been hindered by a lack of consensus about how dropout should be operationalized. The most widely accepted definition in the contemporary dropout literature is based on the therapists’ judgment that the client ended therapy prematurely without their agreement (Warnick et al., 2012). It is acknowledged that dropout can occur after any number of sessions (Wierzbicki and Pekarik, 1993; de Haan et al., 2015), so a strength of this operational definition is that it does not presuppose a treatment duration required to classify a client as a completer or dropout. Another strength is its face validity, as the concept of dropout stems from therapists’ observations that some clients end treatment inappropriately (Wierzbicki and Pekarik, 1993). However, concerns about the reliability of this operational definition have been raised, as it has been acknowledged that therapists may differ in the criteria they use to judge the appropriateness of the ending of treatment (Wierzbicki and Pekarik, 1993). Therefore this approach to defining dropout is subjective, dependent on the clinician’s own views and possibly their therapeutic orientation.

Other definitions of dropout are less subjective. In several studies, dropout was defined based on treatment duration, such that clients are considered to have dropped out if they fail to attend a specific number of sessions or proportion of the planned treatment (Baruch et al., 2009; Warnick et al., 2012). This avoids therapist bias and subjectivity, yet is essentially a dichotomized measure of therapy duration (Hatchett and Park, 2003), which is problematic. Setting a minimum number of sessions does not account for individual differences in how long it takes for a client to benefit from a given treatment, fails to consider the clinical appropriateness of the ending of treatment, and seems inadequate for open-ended therapies, where the number of sessions has not been pre-determined.

Other studies have classified dropouts as clients who do not attend their last scheduled appointment or who repeatedly fail to attend appointments, resulting in no further contact with the therapist (Swift et al., 2009; Warnick et al., 2012). This operationalization is likely to lead to doubtful classifications in several ways. A client who does not schedule another appointment, even though the ending of treatment may have been inadvisable in the therapist’s view, would be classified as a completer (Wierzbicki and Pekarik, 1993). On the other hand, a client who was due to complete treatment, but did not attend their final session, would be classified as a dropout. Moreover, the appropriateness of the treatment ending is not taken into account.

Finally, some studies have defined dropout based on a client ending treatment prior to recovering from the issues that motivated them to seek treatment (Bados et al., 2007; Swift et al., 2009). This approach seeks to provide a more objective judgment on the appropriateness of the ending of treatment, based on clinical outcomes according to standardized outcome measures. However, standardized measures of symptom reduction may not capture the reasons the client sought treatment, or the treatment goals agreed between the client and the therapist. Furthermore, not all clients in psychotherapy and mental health services will return to normal functioning or attain their treatment goals (Edbrooke-Childs et al., 2018).

Thus, currently the most widely accepted definition of dropout in the literature is based upon whether the ending of therapy is mutually agreed between the client and therapist (Wierzbicki and Pekarik, 1993; Hatchett and Park, 2003). Using this definition, a recent meta-analysis of dropout from child and adolescent mental health care estimated the dropout rate in efficacy studies (i.e., randomized controlled trials) at 26%, while average dropout rates were higher (45%) in effectiveness studies conducted in naturalistic settings (de Haan et al., 2013).

It is difficult to estimate the dropout rate specifically in young people receiving therapy for depression, due to the inconsistency in how dropout has been reported. For instance, the TADS trial compared fluoxetine, CBT and their combination for adolescent depression and reported the consent withdrawal rate at 10.9% (Treatment for Adolescents with Depression Study Team, 2004). However, some young people may stop attending treatment without formally withdrawing consent for treatment, which likely explains the difference in the consent withdrawal rate in TADS compared with the dropout rates reported in de Haan et al.’s (2013) meta-analysis. More recently, the “Improving Mood with Psychoanalytic and Cognitive Therapies” (IMPACT) trial investigated psychological treatment for adolescent depression (Goodyer et al., 2011, 2017). In the IMPACT trial, when dropout was defined as ending treatment without the agreement of the therapist, 37% of adolescents were classified as having dropped out of treatment, and a further 11% did not take up the treatment on offer (O’Keeffe et al., in press). Treatment dropout in adolescent depression is an important area for research, given the high dropout rates in this population, and moreover, given that depression is regarded as the leading cause of disability for adolescents (World Health Organization, 2014); yet this is an area that has been neglected in the literature to date.

Kazdin (1996) introduced a risk-factor model of treatment dropout, based on work with children experiencing conduct problems. Risk factors are conditions that are present at the point of intake and cumulatively increase risk of dropout. Studies have generally found the most disadvantaged young people to be at greatest risk of dropout, including those with socio-economic disadvantage, greater parental stress and symptom severity (Kazdin, 1996; de Haan et al., 2013). However, effect sizes are generally small (de Haan et al., 2013) and some studies have found contradictory findings. For instance, some studies have not found symptom severity (Wergeland et al., 2015; O’Keeffe et al., 2018), being from a single parent family (Pina et al., 2003; Gonzalez et al., 2011; Wergeland et al., 2015) and parental wellbeing (O’Keeffe et al., 2018) to be associated with increased risk of dropout. These inconsistent findings may be the result of studies being in different clinical populations or using different definitions of dropout. Although there is some evidence for associations between pre-treatment client characteristics and dropout risk, these are not sufficiently strong to permit reliable prediction of dropout (de Haan et al., 2013). A more diverse range of methods for seeking to improve our understanding of dropout is needed.

The risk-factor model does not consider within-treatment factors, but subsequently Kazdin et al. (1997a,b) developed the barriers to treatment model to address this. This model proposed that families experience multiple barriers when attending treatment which increase the likelihood of them dropping out (Kazdin et al., 1997a,b). Barriers to treatment may include stressors or practical obstacles in attending appointments (such as transportation), not perceiving the treatment as relevant to their problems, finding treatment too demanding or having a poor relationship with their therapist (Kazdin et al., 1997a,b; Nock and Ferriter, 2005). Empirical research has found support for the barriers to treatment model in families attending treatment for a child’s conduct problems, with more reported barriers being associated with greater risk of dropout (Prinz and Miller, 1994; Kazdin et al., 1997b; Kazdin and Wassell, 1998; Stevens et al., 2006). While these studies tell us about issues experienced by families when attending treatment that are associated with dropout, they do not specifically tell us about the reasons families may have for stopping therapy.

Regarding the implications of dropout, it is generally assumed that that dropout is an indicator of treatment failure (Kazdin et al., 1994). Studies with pre-adolescent child and adult clients found dropout to be associated with poorer clinical outcomes (Kazdin and Wassell, 1998; Cahill et al., 2003; Boggs et al., 2005; Saatsi et al., 2007). However, in one study, after pre-treatment differences were controlled for, there was no longer a difference in clinical outcomes between dropouts and completers (Kazdin et al., 1994). Similarly, in the IMPACT trial, no strong evidence was found for poorer outcomes for those adolescents who dropped out of treatment compared with those who completed treatment (O’Keeffe et al., in press). Thus, while dropout is often assumed to be a negative way for therapy to conclude, studies have not always found dropout to be associated with poorer clinical outcomes. This raises questions about the reasons that adolescents stop treatment. Understanding why adolescents stop going to therapy is therefore an important area for research as it can inform clinical practice about the implications of dropout and how disengagement from treatment may be managed.

The limited available literature has focused on parents’ perspectives on the reasons as to why their child dropped out of therapy. Reasons for stopping therapy reported by parents included not perceiving the need for further treatment, the child not liking the clinic and problems in the therapeutic relationship (Kendall and Sugarman, 1997; Garcia and Weisz, 2002). Similarly, in studies with adult clients, reasons for dropping out of treatment include dissatisfaction with the therapy, such as feeling that strategies or advice did not meet their needs, as well as dissatisfaction with the therapist, such as lack of rapport, lack of trust or issues in the fit between the client and therapist (Wilson and Sperlinger, 2004; Roe et al., 2006; Khazaie et al., 2016). One study also reported that clients stopped treatment due to it giving rise to painful feelings or not feeling ready to engage in treatment (Wilson and Sperlinger, 2004). However, positive reasons for stopping treatment have also been cited, with one study of 84 clients finding that almost half reported stopping treatment having made sufficient progress with the problems that led them to seek treatment (Roe et al., 2006). However, no known study has asked adolescents about their reasons for dropping out of therapy, or their therapists about how they make sense of their clients’ decision to stop coming to treatment.

Empirical research into risk factors and within-treatment predictors of dropout has identified some correlates of dropout, but findings from the plethora of studies conducted do not always agree. The views of adolescents on dropout are absent from the literature. There is thus a dearth of knowledge about *why* adolescents drop out of therapy (Ormhaug and Jensen, 2016). Some of the contradictory findings in the literature to date may be the result of issues regarding how dropout is defined, given the limitations of the operational definitions of dropout. In particular, existing definitions of dropout do not take into account the reasons that adolescents give for stopping therapy.

This study therefore aims to identify whether there are more meaningful categories of dropout than the existing dropout definition, based on narratively expressed reasons for dropout given by both therapist and adolescent, in the context of treatment for adolescent depression. The focus is on depression as one of the most commonly occurring presentations for which adolescents seek mental health treatment (Essau, 2005), and among adult clients, dropout rates have been found to be highest for clients with depression (36.4%) compared with other client groups, such as those with anxiety disorders (19.6%) and psychosis (20.1%) (Fernandez et al., 2015). Given the importance of identifying moderators, this study also aimed to test whether there were pre-treatment differences for adolescents in each of the dropout categories, and whether these dropout categories were better at predicting clinical outcomes compared with the existing definition of dropout.

## Materials and Methods

### Design

This study is based on data from the IMPACT trial, a randomized controlled trial comparing three interventions in the treatment of depression in adolescents (Goodyer et al., 2011, 2017). Adolescents (aged 11–17 years) with a diagnosis of moderate/severe unipolar depression were recruited and randomized to a psychological interventions for depression. The multi-center trial was conducted across three regions in the United Kingdom. Four hundred and sixty-five adolescents were recruited and randomized to receive one of the following manualized interventions, in similar numbers (BPI = 155; CBT = 154; STPP = 156):

(i) Brief Psychosocial Intervention (BPI) is a psychosocial program, including a focus on sleep hygiene, exercise and monitoring risk; planned duration of up to 12 sessions delivered over 20 weeks (Kelvin et al., 2010).
(ii) Cognitive-Behavioral Therapy (CBT) focuses on identifying distorted cognitions, and using explicit, shared goals; planned duration of up to 20 sessions delivered over 28 weeks (Impact Study Cbt Sub-Group, 2010).
(iii) Short-Term Psychoanalytic Psychotherapy (STPP) focuses on uncovering the feelings or thoughts that interfere with the young person’s relationships, communication and daily functioning; planned duration of 28 weekly sessions (Cregeen et al., 2016).

Outcome assessments were carried out during treatment (6 and 12 weeks after the start of treatment), post-treatment (36 weeks) and at long-term follow up (52 and 86 weeks).

IMPACT-My Experience (IMPACT-ME) was a qualitative, longitudinal study linked to the IMPACT trial. In the IMPACT-ME study, the participants (including adolescents, parents and therapists) from the North London Centre of IMPACT trial were invited to participate in qualitative interviews about their expectations and experiences of therapy (Midgley et al., 2014).

Drawing on data from the IMPACT and IMPACT-ME studies, the study reported here used a mixed-method, sequential design (Creswell et al., 2003), where qualitative methods were used to construct a typology of dropout, and quantitative methods were then used to investigate whether characteristics and outcomes of adolescents differed between the types of dropout.

### Sample

**FIGURE 1:**
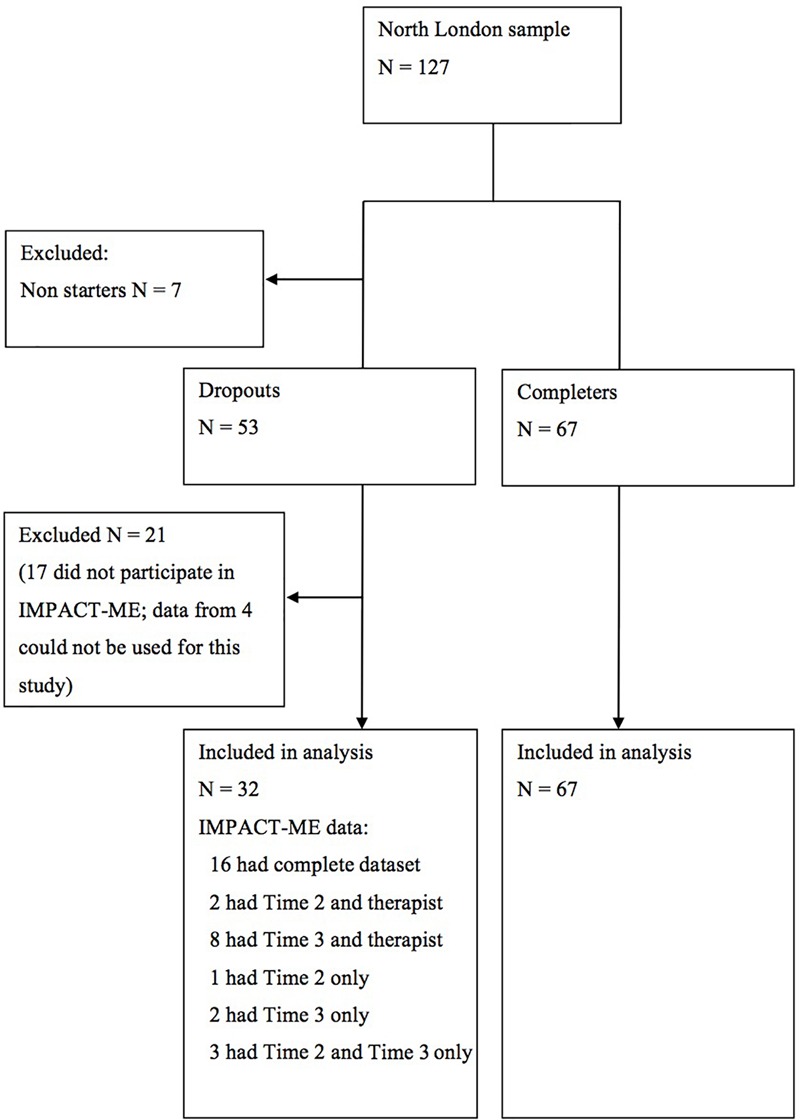
Time 2 refers to the Experience of therapy interview with adolescent (conducted after therapy ended); Time 3 refers to the Thinking back about therapy interview with adolescent (conducted one year after the end of therapy); Complete dataset refers to the adolescent completing a Time 2 and Time 3 interview and their therapist also completing a post-therapy interview.

The sample for this study draws on participants from the North London site of the IMPACT trial (*N* = 127). Of those, seven cases were excluded from this study as they did not take up the therapy on offer and 21 cases who had dropped out of therapy were excluded, either because they did not take part in the IMPACT-ME study (*N* = 17) or because their data could not be used for the purpose of this study (*N* = 4). In such cases, this was because they did not describe their therapy in sufficient detail for them to be classified as a dropout type. The sample for this study thus comprised 99 adolescents from the North London region of the IMPACT trial, 32 of whom dropped out of treatment, while 67 completed treatment (see Figure 1). The 67 completers were not included in the qualitative part of this study, but were used as a comparison group in statistical analyses.

Dropout cases were selected who participated in the IMPACT-ME interviews and were reported as having dropped out of therapy by their therapist. Dropout was defined as the adolescent ending treatment without the prior agreement of their therapist, regardless of when in treatment the ending occurred. For dropout cases, broadly speaking, the sample characteristics appeared similar for those who did and did not participate in the IMPACT-ME study, in terms of average age and depression severity (see Table 1). Although all of those who did not participate in the IMPACT-ME study were female this might be expected as there was a higher prevalence of girls in the sample. The percentages of cases that did and did not participate in the IMPACT-ME study were very similar between the three treatment arms.

**Table 1:** Descriptive statistics for those who dropped out of therapy and did or did not participate in the IMPACT-ME interviews.

	Completed IMPACT-ME	Did not complete IMPACT-ME
	interview (*N* = 36)	interview (*N* = 17)
Age	*M* = 16.02, *SD* = 1.83	*M* = 16.43, *SD* = 1.16
% Female	72%	100%
% White British	49%	64%
MFQ at baseline	*M* = 47.19, *SD* = 1.36	*M* = 47.15, *SD* = 2.62
**Treatment arm**		
BPI	68%	32%
CBT	69%	31%
STPP	67%	33%

*MFQ, Mood and Feelings Questionnaire; BPI, Brief Psychosocial Intervention; CBT, Cognitive Behavioral Therapy; STPP, Short Term Psychoanalytic Psychotherapy.*

The dropout sample for this study comprises the 32 dropout cases where qualitative data was collected and could be used to address the aims of this study (see Figure 1). Of these 32 cases, 9, 9, and 14 participants were in the BPI, CBT and STPP arms, respectively. The sample consisted of 23 females (72%) and 9 males (28%). Their ages at baseline ranged between 11 and 17 years (*M* = 15.84, *SD* = 1.87). Fifteen participants (47%) described their ethnicity as White British, and 16 (50%) described their ethnicity as any other ethnic background (any other white background, mixed, Asian/Asian British, Black/Black British, ant other ethnic group). Ethnicity was unknown for one case.

Adolescents were invited to be interviewed at both time points, and data from both interviews were used in the present study. Not all participants completed both interviews, but available data for each participant was used (see Figure 1). The therapists were not able to be interviewed for six cases, but the therapist interviews were included for all other cases.

### Data

#### Interviews

The data used in this study consisted of interviews with the adolescents and their therapists:

(i) Experience of therapy interviews (Midgley et al., 2011a). Semi-structured interviews were carried out separately with the adolescent and their therapist after the therapy had ended. The interviews with adolescents sought to explore their experiences of therapy and change, including helpful and hindering aspects of therapy and how therapy ended; and interviews with therapists explored the therapy from the clinician’s perspective.
(ii) Thinking back about therapy interviews (Midgley et al., 2011b). Semi-structured interviews were carried out with the adolescent, approximately 1 year after their previous interview, in which their further reflections on the therapy experience were explored.

#### Measures

(i) Depression severity. The Mood and Feelings Questionnaire (MFQ; Angold et al., 1987).
(ii) Anxiety severity. The Revised Children’s Manifest Anxiety Scale (RCMAS; Reynolds and Richmond, 1997).
(iii) Obsessionality. The Short Leyton Obsessional Inventory (LOI; Bamber et al., 2002).
(iv) Anti-social behavior. The Antisocial Behavior Questionnaire (ABQ; St Clair et al., 2017).
(v) Psychosocial functioning. The Health of the Nation Outcome Scale for Children and Adolescents (HoNOSCA; Garralda, 2000).
(vi) Risk taking and self-harm. The Risk-Taking and Self-Harming Inventory for Adolescents (RTSHIA; Vrouva et al., 2010).

### Data Analysis

The aim of this study was to try to identify whether there were more meaningful categories of dropout than the existing definitions allowed for. Ideal type analysis was chosen, as this allows cases to be compared to form clusters of cases, toward the aim of identifying different categories of dropout. The concept of ‘ideal types’ was introduced by Max (Weber, 1949) to describe a composite case that embodied the key attributes of a set of similar cases. Ideal types are defined as a way of representing the characteristics and features of a social phenomenon (Weber, 1949). Ideal types may be thought of as “analytical constructs for use as yardsticks for measuring the similarity and difference between concrete phenomena” (Kvist, 2007). In this context, ‘ideal’ is referring to an idea that presents as a useful way of thinking about clusters of cases, rather than something conceived as perfect (Philips et al., 2007; McLeod, 2011).

As this study was drawing on the perspectives of both adolescents and their therapists, it was expected there would be differences and discrepancies between the accounts given by an adolescent and their therapist. Where their accounts mirrored or contradicted each other became an interesting aspect of the analysis. In the results, the extent to which the account of the adolescent and therapist was similar or different is reported.

Data analysis comprised three key stages: developing the typology, testing the typology; and coding the remaining dataset.

#### Stage 1: Developing the Typology

The typology was initially developed on the first half of the dataset using the stages of ideal type analysis outlined by Gerhardt (1994). This involved listing all themes, categories or statements from the transcript(s) for each case and using this to construct a summary for each case. These summaries were systematically compared with every other case to explore their similarities and differences. Cases were grouped to form discrete types of dropout, whereby each case was represented in one of the types. Cases in each cluster were re-examined, to ensure that they shared key features and did not overlap with other types. A description of each ideal type was written, as well as a coding frame which outlined the necessary conditions that a case must meet to be coded to a type. The typology that was constructed consisted of ideal types, which comprised necessary conditions (i.e., the conditions that a case must meet in order to be coded into that type) and typical characteristics (i.e., characteristics that tended to fit with a type, but were not a requirement to be coded into that type, to reflect variation within the types).

#### Stage 2: Testing the Typology

Two independent researchers used the coding frame to each categorize six cases, using the interview transcripts, into the ideal types. The first was a qualitative post-doctoral researcher, who had experience of ideal type analysis. Agreement with the lead researcher on all but one cases was established. This led to some refinement of the coding frame. A postgraduate researcher without experience of ideal type analysis then used the revised coding frame to code six different cases. There was 100% agreement with the lead researcher on the typological classification. This served as a credibility check for the ideal types.

#### Stage 3: Coding the Remaining Dataset

The coding frame was then used to code the remaining cases. All fitted into the types constructed in the previous stage. Another postgraduate researcher, without experience of ideal type analysis, then double coded all cases that had not yet been double coded, using the coding frame (Table 2). This served as a reliability check, and agreement with the lead researcher was found on all but one case. In the results, the necessary conditions and the typical characteristics are presented, followed by an illustrative case for each type. This provides an example of that type in its optimal form. Where there was significant variation within a type, this is reported in the results.

**Table 2:** Ideal types coding frame.

Type	Summary	Necessary conditions
(1) ‘Dissatisfied’ dropout	The adolescent reported stopping therapy because it failed to meet their needs.	Adolescent reported stopping therapy because they did not find it helpful.
		Adolescent was critical of the therapy they received.
		Therapist reported that adolescent had difficulty attending or engaging in the sessions.
(2) ‘Got-what-they-needed’ dropout	The adolescent reported stopping therapy because they felt better.	Adolescent reported not seeing a need to keep going to therapy, as they felt better or it was due to end soon.
		Adolescent attributed positive change, to some extent, to the therapy.
		Therapist did not appear to be worried about the adolescent stopping therapy.
(3) ‘Troubled’ dropout	The adolescent reported stopping therapy because they felt it was not the right time for them to engage in therapy.	Adolescent presented with complex difficulties (e.g., homelessness, history of abuse)
		Adolescent linked (or implied) stopping therapy to external difficulties.
		Therapist suggested that the adolescent could not have engaged in any type of therapy at that time, because of the lack of stability in their life.

#### Stage 4: Quantitative Analysis

Having constructed the types of dropout, Kruskal–Wallis tests were conducted to test whether there were differences between the cases in each dropout type and completers with respect to baseline characteristics. Where the Kruskal–Wallis test statistic was statistically significant (*p* < 0.05), *post hoc* pairwise comparisons were conducted using Dunn’s tests, with Benjamini–Yekutieli adjustment for multiple comparisons to control the false discovery rate (Benjamini and Yekutieli, 2001). Hypotheses about differences in clinical outcomes between the dropout types were formed. The final stage of data analysis was to test whether there was a difference in outcomes between the types. Mixed effect models were used to test differences between MFQ scores for each type at baseline, long-term follow-up, and change over time. The dependent variable was MFQ scores, as this was the primary outcome measure in the IMPACT RCT (Goodyer et al., 2011). The independent variables were Time × Therapy Ending Type interaction effects, with the types included as categorical variables. Three models were tested: predicting change in MFQ scores at 36, 52, and 86 weeks in Stata version 14.1. Models included a random intercept and random slope for participant, and a random intercept for therapist.

### Ethics Statement

The study protocol was approved by Cambridgeshire 2 Research Ethics Committee (Reference: 09/H038/137). Fully informed written consent was sought from participants at the baseline assessment. For those under the age of 16, fully informed written parental consent was also sought. To ensure the confidentiality of participants, participants were assigned a pseudonym and any identifiable details have been removed or changed.

## Results

Three types of dropout were constructed, using ideal type analysis: ‘dissatisfied’ dropout, ‘got-what-they-needed’ dropout and ‘troubled’ dropout. In the BPI arm, the ‘got-what-they-needed’ type was most common, with five cases fitting into this type. The remaining BPI cases were classified as ‘dissatisfied’ (*N* = 3) and ‘troubled’ (*N* = 1). As in the BPI arm, the most common type in the CBT arm was the ‘got-what-they-needed’ type, with four CBT dropouts fitting this type. The remaining CBT cases were ‘dissatisfied’ (*N* = 3) and ‘troubled’ (*N* = 2). In the STPP arm, the most common type was the ‘dissatisfied’ type, with twelve STPP dropouts fitting this type. Of the remaining two STPP dropouts, one was classified as a ‘got-what-they-needed’ dropout and one as a ‘troubled’ dropout.

### Ideal Type 1: ‘Dissatisfied’ Dropout

#### Description

‘Dissatisfied’ dropouts reported stopping therapy because they did not find therapy helpful and it failed to meet their needs. Eighteen cases represented this type (BPI = 3, CBT = 3, STPP = 12).

#### Necessary Conditions

‘Dissatisfied’ dropouts were critical of the therapy they received and described various things about the therapy they did not like or find helpful, such as the therapists’ approach to therapy, and issues regarding their relationship with their therapist. They reported stopping therapy because they did not feel they were benefitting from it. The therapist of ‘dissatisfied’ dropouts reported that the adolescent showed some reluctance to engage, either in the sessions, or through missed sessions.

#### Typical Characteristics

‘Dissatisfied’ dropouts may have referred to practical issues associated with attending therapy, but did not cite these as reasons for stopping therapy. They sometimes spoke about not feeling able to tell their therapist how they felt about therapy, particularly the aspects of therapy they were dissatisfied with. Their therapists tended to report that they believed the ending of therapy was the result of the adolescents’ inability to engage in the therapy. The therapists appeared to be unaware of many of the adolescents’ criticisms of therapy. Their narrative of the therapy therefore tended to be distinctly different from that of the adolescents.

#### Significant Variation

While in all three treatment arms, adolescents expressed dissatisfaction with the therapy, there were differences in the nature of their dissatisfaction. In the BPI and CBT arms, adolescents described dissatisfaction with the therapy being too structured or not understanding the rationale for some of the activities in therapy, such as keeping a diary. In contrast, dissatisfaction in the STPP arm tended to focus on the lack of structure, not knowing what to talk about, feeling uncomfortable with silence in the sessions or the therapist offering interpretations that didn’t make sense to them.

#### Illustrative Case: Fiona

Fiona was a 13-year-old girl who received STPP.

##### Adolescent’s perspective

Fiona was critical of the therapy she received. Fiona’s main criticism was with the way in which the therapist interacted with her. She described how the therapist would ask her questions, but when she answered, the therapist wouldn’t respond, and they could spend 5 minutes in silence, which Fiona described as “awkward.” Fiona described her therapy:

“I went to this therapist and they just sat there and hummed for an hour at everything that I said. I hated it. [My therapist] made me really angry because it just felt like I was talking to a brick wall and I wasn’t. I didn’t even want to talk because [my therapist] didn’t engage with me at all. It just felt like it was completely pointless.”

Fiona described finding the therapy “disappointing” and also reported not feeling comfortable telling the therapist how she felt. Fiona described how her decision to stop going to therapy came about:

“Well I wasn’t enjoying it, well not enjoying it because it’s not something you’re going to have fun in doing, but I wasn’t benefiting from it and it just seemed really pointless because it was quite far away and I didn’t feel like I was getting anything out of it. And I was missing time off school to actually get there on time.”

While Fiona referred to the inconvenience of attending therapy, she implied this was not the reason for stopping: therefore, it is possible she may have kept going, had she felt she was benefitting from it.

##### Therapist’s perspective

The therapist reported that at the start of therapy, Fiona had expressed reservations about therapy. Despite this, the therapist described seeing a side to Fiona that could engage in the therapy, as she was at times “animated,” but she then felt Fiona withdraw. The therapist reported that Fiona then said she did not want to continue with therapy. The therapist speculated that things had already started to improve for her at an early stage in the therapy and the therapist suggests this may have impacted on her willingness to engage:

“I think the session sort of stirred stuff up and the fear was that she’d feel worse again.”

The therapist reported that Fiona believed she was better when she decided to stop therapy, whereas the therapist stated that they did not believe things were truly resolved for Fiona.

### Ideal Type 2: ‘Got-What-They-Needed’ Dropout

#### Description

‘Got-what-they-needed’ dropouts reported stopping therapy because from their perspective, they had got what they needed and did not feel a need to continue in therapy. Ten cases represented this type (BPI = 5, CBT = 4, STPP = 1).

#### Necessary Conditions

‘Got-what-they-needed’ dropouts appeared to find therapy helpful and attributed positive change in their life, at least to some extent, to the therapy they received. They reported their reason for stopping therapy to be that they felt they had got the help they needed. The therapists likewise reported that they thought their clients had got what they needed from therapy but viewed the ending as premature in that they believed continued therapy could have yielded further benefits. The therapist did not appear to be left clinically concerned about ‘got-what-they-needed’ dropouts, as they reported seeing some improvements for the adolescent by the time therapy ended.

#### Typical Characteristics

‘Got-what-they-needed’ dropouts may have been critical of specific aspects of the therapy or may have referred to the inconvenience of attending sessions, but did not cite these as reasons for stopping therapy. The therapists tended to report signs of disengagement for ‘got-what-they-needed’ dropouts, either through missing sessions or their reluctance to engage when they did attend.

#### Illustrative Case: Connor

Connor was a 17-year-old boy, who received CBT.

##### Adolescent’s perspective

Connor gave a balanced account of his therapy, as he discussed aspects he found positive about it, as well as some criticisms of the therapy. Connor reported that it was “helpful to talk to someone.” He spoke positively about his therapist and the relationship they had:

“[My therapist] wanted to help. Not judgmental or anything. You know, like a nice person. So it was a good relationship.”

Connor also spoke about some reservations regarding the approach to therapy, as he questioned “why can’t we just talk about stuff?” instead of focusing on a specific goal. Overall, Connor gave the impression that he had got something out of the therapy, despite his reservations. Connor linked his decision to stop therapy to external circumstances. He suggested that the main trigger to his depression was school, and once he finished school, he reported feeling ready to stop therapy:

“I just wanted to kind of, get that kind of phase of my life over with. I didn’t really want to, like, it was almost like doing the stuff put me in a worse mood, because it would put me in a mind-set of, oh ok, I’m going to a therapy meeting now, that means I have, something to talk about, about why I’m feeling bad.”

Connor described feeling better by this point, so reported not feeling a need to continue with therapy.

##### Therapist’s perspective

Connor’s therapist described him as compliant with the treatment, in that he attended most of the sessions, although also described how he seemed “reluctant” to be there. The therapist described how they focused on Connor’s sleep patterns in the sessions, and reported that this seemed helpful for Connor. Connor’s therapist described how Connor “stopped coming” to therapy, and connected this to his ambivalence toward therapy. However, the therapist reported that Connor had benefitted from therapy by the time he decided to stop, and did not seem concerned about him ending therapy, despite not agreeing to the ending. The therapist suggested that the practical level of support that therapy offered him seemed to be the right approach for him, at that point in his life, yet speculated that Connor may need more therapy in the future.

### Ideal Type 3: ‘Troubled’ Dropout

#### Description

‘Troubled’ dropouts reported stopping therapy because of a lack of stability in their life which made it difficult for them to engage in therapy. Four cases represented this type (BPI = 1, CBT = 2, STPP = 1).

#### Necessary Conditions

‘Troubled’ dropouts described significant difficulties beyond their low mood (including homelessness, history of abuse and trauma, and financial and caring responsibilities). ‘Troubled’ dropouts and their therapists gave similar accounts; both described how a lack of stability in the adolescent’s life impacted on their session attendance and led to their decision to stop therapy. The therapists suggested this lack of stability needed to be addressed before these adolescents would be able to engage in therapy.

#### Typical Characteristics

The therapists of ‘troubled’ dropouts tended to report that the adolescents engaged in the sessions when they attended, but they missed a lot of sessions, as a result of the external difficulties in their lives. The therapists suggested these external difficulties were the main reasons for them stopping therapy.

#### Significant Variation

‘Troubled’ dropouts varied in how they spoke about their experience of therapy. While some reported not finding it helpful, others spoke about finding aspects of it helpful, such as being offered advice and the relief of talking to someone. Regardless of whether ‘troubled’ dropouts spoke about therapy being helpful or unhelpful, they did not tend to link this to their decision to stop therapy.

#### Illustrative Case: Asha

Asha was a 17-year-old girl, who received BPI.

##### Adolescent’s perspective

Asha described how she initially attended the therapy sessions, but then decided to stop going:

“I went for a while and then and then [sic] I just stopped going. Just because I felt like I wasn’t changing anything and my life was all over the place and I just like oh, yeah, just stopped going.”

While Asha described stopping therapy because she didn’t feel she was gaining from it, she also linked it to external factors in her life, suggesting that the complex difficulties made it difficult for her to engage in therapy, as she did not have a stable home.

##### Therapist’s perspective

Asha’s therapist reported that Asha’s therapy attendance had been “intermittent.” The therapist linked Asha’s difficulty attending the sessions to demands in her home life, and reported that the focus of the sessions was on helping Asha to manage her living situation. The therapist speculated that with the instability in her life, Asha may not have been able to engage in any kind of treatment:

“So I’m not sure, you know, as far as an individual therapy is concerned, whether that, whether anything would’ve worked at that time.”

Therefore, the therapist seemed doubtful that any talking therapy could have worked at that point in Asha’s life, and suggested that Asha needed to find stability in her life before she could attend treatment regularly.

### Comparison of the Cases in the Ideal Types

Having constructed a typology of dropout, further exploration of the types was conducted, comparing the cases in the ideal types. This was to test whether the refined categorization of dropout was more meaningful compared with the generic ‘dropout’ definition in identifying baseline characteristics associated with dropout and association with outcome. There was an insufficient sample size to conduct statistically reliable analyses for ‘troubled’ dropouts with respect to clinical outcomes. However, some specific hypotheses regarding ‘got-what-they-needed’ dropouts and ‘dissatisfied’ dropouts were formed, and there was a sufficient sample size to conduct statistical analyses comparing ‘got-what-they-needed’ dropouts and ‘dissatisfied’ dropouts with those who completed therapy.

#### Hypotheses

(i) ‘Got-what-they-needed’ dropouts will have been less severely depressed at baseline, compared with ‘dissatisfied’ dropouts.
(ii) ‘Got-what-they-needed’ dropouts will have had better outcomes, compared with ‘dissatisfied’ dropouts.
(iii) ‘Got-what-they-needed’ dropouts will have had better outcomes, compared with completers.
(iv) ‘Dissatisfied’ dropouts will have had poorer outcomes, compared with completers.

The first hypothesis was formed on the basis that ‘got-what-they-needed’ dropouts may have been less severely depressed to begin with than ‘dissatisfied’ dropouts and therefore required a brief number of sessions to feel sufficiently improved to stop therapy. The hypotheses regarding outcomes were formed on the basis that ‘got-what-they-needed’ dropouts reported finding therapy helpful and ‘dissatisfied’ dropouts reported finding therapy unhelpful, so it was expected that ‘dissatisfied’ dropouts would have poorer outcomes than ‘got-what-they-needed’ dropouts. As the completers had not been grouped into types, it was expected that they would comprise a heterogeneous group. It was therefore expected that completers would have poorer outcomes compared with ‘got-what-they-needed’ dropouts and better outcomes than ‘dissatisfied’ dropouts.

#### Comparison of Pre-treatment Characteristics for Completers, ‘Dissatisfied’ Dropouts, ‘Got-What-They-Needed’ Dropouts and ‘Troubled’ Dropouts

Baseline descriptive statistics are shown in Table 3 for adolescents for each dropout category and completers, to explore whether there were differences between the dropout types and completers. Kruskal–Wallis tests indicated that there was not a statistically significant difference (at the 5% level of significance) between the dropout types and completers with respect to age, depression and anxiety severity, obsessionality, psychosocial functioning and self-harm. There was a statistically significant difference in antisocial behavior between the groups (Kruskal–Wallis χ^2^ = 13.85, *p* = 0.003). Based on Dunn’s pairwise tests with Benjamini–Yekutieli adjustment for multiple comparisons, completers were found to have statistically significantly lower scores of antisocial behavior at baseline compared with ‘got-what-they-needed’ dropouts (*p* = 0.03) and ‘troubled’ dropouts (*p* = 0.04). All other pairwise comparisons of anti-social behavior baseline scores yielded *p*-values larger than 0.05.

**Table 3:** Baseline descriptive statistics for dropout types and completers.

	Completers		‘Got-what-they-needed’	‘Dissatisfied’		‘Troubled’
	*N* = 67		dropouts *N* = 10	dropouts *N* = 18		dropouts *N* = 4
Sex (% female)	69%		60%	72%		100%
Ethnicity (% White British)	59%		40%	65%		0%
Comorbidity (% with >1 comorbid disorder)	48%		50%	33%		100%

					**Kruskal–Wallis**
	***M* (*SD*)**	***M* (*SD*)**	***M* (*SD*)**	***M* (*SD*)**	**χ^2^ (*df* = 3)**	***p*-value**

Age	15.63 (1.63)	14.97 (1.82)	16.12 (1.95)	16.73 (0.65)	4.69	0.20
Depression (MFQ)	45.69 (11.32)	47.12 (6.21)	47.67 (9.72)	45.98 (6.16)	0.53	0.91
Anxiety (RCMAS)	41.47 (7.68)	44.66 (5.89)	40.37 (7.20)	44.50 (3.11)	2.61	0.46
Obsessionality (LOI)	10.77 (5.25)	10.81 (5.08)	9.78 (5.55)	8.20 (3.58)	1.35	0.72
Antisocial Behavior (ABQ)	2.95 (2.66)	5.50 (2.80)	3.67 (2.06)	8.00 (4.24)	13.85	0.003
Psychosocial functioning (HoNOSCA)	18.55 (6.63)	15.55 (6.29)	20.90 (7.88)	21.11 (6.19)	3.32	0.35
Risk taking (RTSHIA)	5.13 (5.04)	5.25 (4.20)	6.77 (4.83)	12.75 (4.03)	8.47	0.04
Self-harm (RTSHIA)	11.24 (8.71)	12.68 (7.64)	17.97 (12.92)	17.81 (11.89)	4.87	0.18

*M* = *mean; SD* = *standard deviation; MFQ* = *Mood and Feelings Questionnaire; RCMAS* = *Revised Children’s Manifest Anxiety Scale; LOI* = *Leyton Obsessional Inventory; ABQ* = *Antisocial Behaviors Questionnaire; HoNOSCA* = *Health of the Nation Outcomes Scales Child and Adolescent; RTSHIA* = *Risk Taking and Self Harm Inventory.*

The Kruskal–Wallis test also indicated a statistically significant difference between groups in baseline scores of risk taking (Kruskal–Wallis χ^2^ = 8.47, *p* = 0.04), although Dunn’s pairwise tests found no statistically significant difference between any pair of groups (at the 5% level of significance). All ‘troubled’ dropouts presented with at least one comorbid disorder, whereas comorbidity rates were lower in the other three groups. Statistical testing comparing groups and rates of comorbidity was not conducted due to the presence of zero values in some cells, which meant it was not possible to conduct chi-squared tests. This was also the case for the other categorical variables. Overall, the ‘troubled’ dropouts seemed to present with more difficulties at baseline, especially compared with the completers.

#### Testing Outcomes for Completers, ‘Dissatisfied’ Dropouts and ‘Got-What-They-Needed’ Dropouts

**FIGURE 2:**
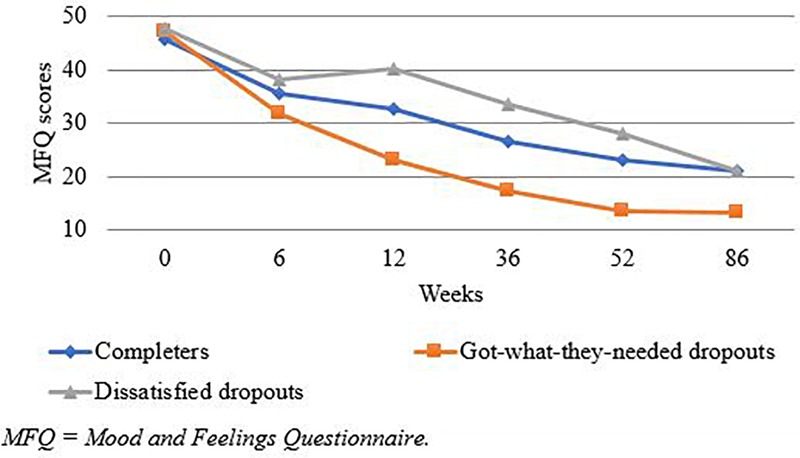
Mean MFQ scores at each time point, for ‘got-what-they-needed’ dropouts, ‘dissatisfied’ dropouts and completers.

Figure 2 shows the mean MFQ scores at each time point, for ‘got-what-they-needed’ dropouts, ‘dissatisfied’ dropouts and completers. MFQ scores reduced for all groups over time, with ‘got-what-they-needed’ dropouts making the greatest gains. Hypotheses were tested using mixed effects models, with MFQ scores as the dependent variable, and Time × Therapy Ending Type interaction effects as the independent variables. Therapy ending type was coded as dummy variables for completers, ‘got-what-they-needed’ dropouts, and ‘dissatisfied’ dropouts. The model statistics are presented in Table 4.

**Table 4:** Mixed effect models predicting MFQ scores from Time and Therapy Ending Type, with completers as the reference group.

	Model 1	Model 2	Model 3
	36 weeks	52 weeks	86 weeks
Variable	β (*SE*)	β (*SE*)	β (*SE*)
Constant	45.69 (1.28)	45.69 (1.28)	45.69 (1.28)
Time	-19.51^∗^ (2.07)	-22.52^∗^ (2.25)	-25.39^∗^ (1.97)
Group (reference: completers)			
‘Got-what-they-needed’ dropouts	1.44 (3.55)	1.44 (3.55)	1.44 (3.55)
‘Dissatisfied’ dropouts	1.98 (2.78)	1.98 (2.78)	1.98 (2.78)
Time × ‘got-what-they-needed’ dropouts	-10.37 (5.62)	-10.93 (6.55)	-8.91 (5.43)
Time × ‘dissatisfied’ dropouts	5.41 (4.89)	3.17 (4.82)	-1.87 (4.27)
Residual variance	90.66	101.40	82.56
Participant variance	5.63	0.67	12.13
Participant slopes	7.63	6.84	2.78
Therapist variance	5.66	0.70	12.10

*MFQ, Mood and Feelings Questionnaire. ^∗^ < 0.001.*

The estimated MFQ scores at each time point are presented in Table 5. No evidence was found for a significant difference in depression severity at baseline between completers, ‘got-what-they-needed’ dropouts and ‘dissatisfied’ dropouts. Thus, the first hypothesis that ‘got-what-they-needed’ dropouts would be less severely depressed at baseline compared to ‘dissatisfied’ dropouts was not supported.

**Table 5:** Estimated mean MFQ scores at 36, 52, and 86 weeks, showing group comparisons for completers, ‘dissatisfied’ dropouts and ‘got-what-they-needed’ dropouts.

	Completers *N* = 67	‘Dissatisfied’ dropouts *N* = 18	‘Got-what-they-needed’ dropouts *N* = 10	Completers vs. ‘dissatisfied’ dropouts	Completers vs. ‘got-what-they-needed’ dropouts	‘Dissatisfied’ dropouts vs. ‘got-what-they-needed’ dropouts
Weeks	Mean (*SE*)	Mean (*SE*)	Mean (*SE*)	*p*-value	*p*-value	*p*-value
0	45.69 (1.55)	47.67 (2.99)	47.12 (4.01)	0.48	0.69	0.90
36	26.17 (1.67)	33.56 (3.62)	17.24 (4.21)	0.27	0.07	0.02
52	23.17 (1.75)	28.32 (3.32)	13.68 (4.82)	0.51	0.10	0.06
86	20.30 (1.62)	20.40 (3.10)	12.82 (4.14)	0.66	0.10	0.26

*Estimates and group comparisons derived from mixed effect models predicting MFQ scores from Time and Therapy Ending Type.*

In line with the hypotheses, the greatest improvement was observed for ‘got-what-they-needed’ dropouts, followed by completers, with ‘dissatisfied’ dropouts having the poorest outcomes, at 36, 52, and 86 weeks. At 36 weeks, the hypothesis that ‘got-what-they-needed’ dropouts would have better outcomes compared with ‘dissatisfied’ dropouts was supported, although there was not a statistically significant difference between the two groups at the later follow-ups (Table 5).

Despite trends in the expected direction, the hypothesis that ‘got-what-they-needed’ dropouts would have better outcomes compared with completers was not supported, as there was not a statistically significant difference between the two groups at any time point. Similarly, despite trends in the expected direction for ‘dissatisfied’ dropouts compared with completers, there was not a statistically significant difference between the two groups at any time point.

## Discussion

This study aimed to identify categories of dropout that, in contrast to previously proposed definitions of dropout, took into account the perspective of both the client and their therapist. A further aim was to test whether this refined categorization of dropout was better at predicting clinical outcomes than the generic ‘dropout’ definition, in adolescents who received therapy for depression. Three distinct types of dropout were constructed. ‘Got-what-they-needed’ dropouts were those who reported stopping therapy because they felt better. ‘Dissatisfied’ dropouts were those who reported stopping therapy because they did not find it helpful. ‘Troubled’ dropouts reported stopping therapy because of a lack of stability in their lives that made it difficult for them to engage in therapy.

‘Got-what-they-needed’ dropouts reported that they did not perceive a need to continue in therapy and their therapists were not left concerned about them. The ‘got-what-they-needed’ dropout category fits with qualitative studies that cite clients reporting not perceiving the need for further treatment as a reason for stopping treatment (Garcia and Weisz, 2002; Block and Greeno, 2011). A substantial minority of cases in this sample (31%) were ‘got-what-they-needed’ dropouts, suggesting that adolescents stopping therapy without agreement of their therapist is not necessarily a negative way for therapy to conclude. While we could speculate these adolescents were justifying their decision to end therapy by saying they didn’t need to keep going, this study found a trend toward them having better outcomes compared with ‘dissatisfied’ dropouts and completers, supporting their reported perception that they did not need to continue in therapy. However, the study was underpowered and there was only a statistically significant difference between ‘got-what-they-needed’ dropouts and ‘dissatisfied’ dropouts at 36 weeks with regards to depression severity – but not at the later follow ups. This finding must be viewed cautiously but may suggest a direction for future research to rigorously test the link between dropout types and clinical outcomes in a sufficiently powered study. Importantly, baseline scores indicated that ‘got-what-they-needed’ dropouts did not appear to be less severely depressed compared with completers or ‘dissatisfied’ dropouts. These findings suggest that a significant minority of adolescents with moderate to severe depression may benefit from a brief intervention and be able to decide to end therapy appropriately, even when this has not been agreed with the therapist. Their therapists viewed the ending as premature, yet did not have clinical concerns about these adolescents. Overall, ‘got-what-they-needed’ dropouts appeared to have stopped therapy for positive reasons, in contrast to the other types of dropout.

‘Dissatisfied’ dropouts were critical of the therapy they received, and described a range of things they didn’t like about the therapy or that they found unhelpful, including issues with the therapists approach and their relationship with the therapist. ‘Dissatisfied’ dropout is consistent with some aspects of the barriers to treatment model, which outlines difficulties experienced by families in attending treatment (Kazdin et al., 1997a,b). Such difficulties include perceptions that treatment is not relevant or is too demanding and issues in the relationship with the therapist, which are particularly relevant to ‘dissatisfied’ dropouts. ‘Dissatisfied’ dropouts frequently referred to practical issues in attending therapy, such as the cost of bus fares, which fit with ‘obstacles to coming to therapy’ from the barriers to treatment model. Research has found more obstacles experienced by families to be associated with greater risk of dropout (Prinz and Miller, 1994; Kazdin et al., 1997a,b; Kazdin and Wassell, 1998; McCabe, 2002; Stevens et al., 2006). However, ‘dissatisfied’ dropouts did not cite practical issues as reasons for stopping therapy. Rather, for these adolescents, the costs of therapy seemed to outweigh the benefits. Thus, it seems that adolescents’ perceived lack of helpfulness of treatment was central to their decision to stop treatment. At baseline, there did not appear to be any notable differences between ‘dissatisfied’ dropouts and the completers with regards to presenting symptoms, indicating of the measured variables, there were not factors that could have predicted the outcome of ‘dissatisfied’ dropout.

Therapists of ‘dissatisfied’ dropouts showed little awareness of the adolescent’s dissatisfaction with treatment, which fits with previous findings that clients often avoid expressing their dissatisfaction to their therapist (Henkelman and Paulson, 2006; von Below and Werbart, 2012; Gibson and Cartwright, 2013). This mirrors what was found in our study, as the adolescents expressed many criticisms of therapy in the research interviews, yet often did not seem to have shared these criticisms with their therapists, with some adolescents explicitly stating that they did not feel comfortable expressing their negative views about therapy to their therapist.

‘Troubled’ dropouts reported stopping therapy because of a lack of stability in their lives, which made it difficult for them to engage in the therapy, at that time. These adolescents reported complex difficulties that had interfered with therapy (such as not having a stable home or having responsibilities to support their family). Moreover, at baseline, ‘troubled’ dropouts appeared the most impaired in terms of symptom severity, compared with the other dropout types and completers. This included having statistically significantly higher scores for antisocial behavior, compared with completers, as well as presenting with more comorbidity. This type fits with Kazdin’s (1996) ground-breaking risk-factor model, which suggests that it is the most disadvantaged youth at greatest risk of dropping out of treatment (Kazdin, 1996; de Haan et al., 2013). ‘Troubled’ dropouts most certainly would have met the criteria for a number of risk factors, and therefore according to Kazdin’s risk-factor model, would have been considered at high risk of dropout. A recent systematic review revealed that intercurrent life events and contextual factors that interfere with treatment have been largely overlooked in the child psychotherapy literature (Blackshaw et al., 2018). ‘Troubled’ dropouts represent a group of young people for whom there were contextual factors that impeded their ability to engage in treatment, reflecting the need for greater attention to be paid to such contextual complexity for delivering effective mental health care. The reasons ‘troubled’ dropouts reported for stopping therapy focused on issues outside of the therapy room, contrasting with the other types of dropout, whose reasons for stopping therapy centered around what happened in the therapy and whether or not they found it helpful.

Kazdin’s (1996) risk-factor model has received a great deal of attention in the literature on treatment dropout. While we must be cautious about the claims that can be made from our small sample, the risk-factor model appeared relevant to ‘troubled’ dropouts, but not the other types of dropout in this study. It is possible that the risk-factor model is primarily important for understanding one type of dropout only, and may be less helpful in explaining other types of dropout (‘dissatisfied’ and ‘got-what-they-needed’ dropouts), who appeared similar to completers prior to the start of treatment. Within-treatment factors may be a more productive line of enquiry for understanding ‘dissatisfied’ dropouts among adolescents in therapy for depression, while ‘got-what-they-needed’ dropouts reflect cases that drop out of treatment for more positive reasons. Together, these findings illustrate issues when using the generic ‘dropout’ definition. Future research should use a more refined categorization of dropout, due to the heterogeneity of cases classified as dropouts when using existing definitions of dropout.

Of the dropout cases included in this study, the most common type of dropout in the BPI and CBT arms was ‘got-what-they-needed’ dropout, with 42% and 45% of dropouts in these treatments fitting with this type. This finding may be understood in the context of the BPI and CBT treatment models, which focus on the presenting symptoms, which may have resulted in early symptom relief, resulting in these adolescents considering themselves to be sufficiently improved to stop therapy. The most common type in the STPP arm was ‘dissatisfied’ dropout, with 79% of STPP dropouts fitting with this type, compared to 25% of BPI and 33% of CBT cases. This raises questions about the specific aspects of STPP that adolescents seemed particularly dissatisfied with. These included the adolescents disliking the lack of structure, not knowing what to talk about and finding silence uncomfortable. Therapists may need to look out for warning signs of disengagement, and in some cases, aspects of the STPP model may need to be adapted to better meet their needs.

### Strengths and Weaknesses of This Study

This mixed-methods study allowed an in-depth exploration of the concept of dropout. The qualitative analysis was strengthened by credibility and reliability checks in developing the types. However, there were too few cases to allow comparison of ‘troubled’ dropouts with other groups with respect to outcome, and the sample size for ‘got-what-they-needed’ and ‘dissatisfied’ types meant the statistical analyses had low power to detect differences in both baseline characteristics and outcome. There were too few cases to control for potential confounders, and the length of time between the end of treatment and the follow up assessments varied between participants. Thus, the statistical analyses were exploratory and failure to reject the null hypothesis should not be interpreted as evidence that the groups did not differ with respect to clinical outcomes. We hope that larger studies in the future will build on these exploratory results. As participants had been randomized to a treatment arm, the method of treatment assignment was not naturalistic, so dropout could potentially have been the result of violation of client preferences for the type of treatment, although none of the participants stated that they stopped therapy for this reason. Additionally, the study sample comprised adolescents with depression. It is unknown how generalizable these findings are to adolescents with other presenting problems. Future studies can test how these types apply to naturalistic settings and with adolescents with other presenting difficulties.

We also note that the sample for this study comprised those adolescents and therapists who were contactable and agreed to be interviewed after the therapy ended, so it is unknown whether these types would generalize to those who did not participate in the study. This study used semi-structured interviews, which provided a rich account of the participants’ experiences of therapy, yet there may be bias in what was reported. The data used in this study was based on what the participants were able to remember, willing to share and aware of. It is possible that there may have been reasons for dropout that the adolescent and therapist were not aware of or had forgotten by the time they were interviewed. Finally, ideal type analysis shares limitations with many inductive analyses of qualitative data. The types identified in this study may not be the only types of dropout, and other types may be found in other samples and settings. The typology was constructed from the first author’s point of view, as a researcher. It cannot be said whether the same types would have been constructed by another researcher. Nonetheless, once the typology was defined, there was good agreement in classification of cases to the types between the lead author and independent researchers.

## Conclusion

Debates about how dropout should be defined have spanned across several decades. The aim of this study was to try to identify more meaningful categories of dropout in the context of adolescents receiving psychological therapy for depression. In this study, three types of therapy dropout were constructed. While the adolescents decided to stop therapy without their therapists’ agreement, they had somewhat different reasons for doing so and reported several key influences as to whether they kept going to therapy: whether the therapy was helping or had helped them, their satisfaction with the treatment and external influences.

‘Dissatisfied’ dropouts had significantly poorer outcomes compared with ‘got-what-they-needed’ dropouts at 36 weeks. The study had low statistical power and these findings should be viewed as preliminary, yet provide some indication that the effect of dropout on outcome may differ by dropout type. This study raises issues with studying dropout as a unitary concept and may help to explain some of the inconsistent findings in the existing dropout literature. Existing definitions of dropout do not capture or take into account the way in which adolescents experience therapy, nor do they consider the reasons they give for stopping therapy. Future research should seek to differentiate between different types of dropout given the heterogeneity of cases when using the generic ‘dropout’ definition.

The types of dropout in this study may provide a framework for clinicians working in CAMHS to think about ending treatment with adolescents receiving therapy for depression. ‘Got-what-they-needed’ dropouts may to a certain extent be thought of as having dropped out of therapy appropriately, given that the adolescents reported that they did not perceive a need to continue in treatment. Dropping out of therapy may not always be a negative way for therapy to end, so in clinical practice, shared decision making (Cheng et al., 2017) about treatment durations and endings may be warranted. ‘Dissatisfied’ dropouts reported stopping therapy because of issues they had with the therapy. These findings are important for providing awareness to clinicians about the range of issues experienced by adolescents in treatment that lead to their dissatisfaction and disengagement. Through awareness of such issues, therapists can be more in tune with the way in which adolescent’s experience treatment, and interventions can be adapted to improve their acceptability to adolescents. Therapists often were not aware of the issues adolescents had with treatment. Future research into the therapeutic process should seek to investigate whether there are detectable warning signs of adolescents’ dissatisfaction with treatment. Finally, the ‘troubled’ dropouts illustrate the difficulty some adolescents are likely to have engaging in treatment when experiencing complex difficulties, such as homelessness or responsibilities in the family. This raises questions about how such adolescents, possibly those most in need of it, can be supported.

## Author Contributions

NM and MT were Principal Investigators of the IMPACT-ME study, responsible for securing funding for the project and the overall management of the project. SO’K contributed to data collection. SO’K carried out this study, including analyzing the data and writing the initial draft of this manuscript, under the supervision of NM and PM. All authors contributed to manuscript revision, read and approved the submitted version.

## Conflict of Interest Statement

The authors declare that the research was conducted in the absence of any commercial or financial relationships that could be construed as a potential conflict of interest.
